# Shedding Light on the Chemical Diversity of Ectopic Calcifications in Kidney Tissues: Diagnostic and Research Aspects

**DOI:** 10.1371/journal.pone.0028007

**Published:** 2011-11-18

**Authors:** Arnaud Dessombz, Dominique Bazin, Paul Dumas, Christophe Sandt, Josep Sule-Suso, Michel Daudon

**Affiliations:** 1 Laboratoire de Physique des Solides, Bat. 510, Université Paris Sud, Orsay, France; 2 Synchrotron SOLEIL, L'Orme des Merisiers, Saint-Aubin - BP 48, Gif-sur-Yvette, France; 3 Cancer Centre, University Hospital of North Staffordshire, Newcastle Road, Stoke-on-Trent, Staffordshire, United Kingdom; 4 AP-HP, Hôpital Tenon, Service d'Exploration Fonctionnelle, Paris, France; Universidade de Sao Paulo, Brazil

## Abstract

In most industrialized countries, different epidemiologic studies show that chronic renal failure is dramatically increasing. Such major public health problem is a consequence of acquired systemic diseases such as type II diabetes, which is now the first cause for end stage renal failure. Furthermore, lithogenic diseases may also induce intratubular crystallization, which may finally result in end-stage renal failure (ESRF). Up to now, such rare diseases are often misdiagnosed. In this study, based on twenty four biopsies, we show that SR µFTIR (Synchrotron Radiation-µFourier transform infrared) spectroscopy constitutes a significant opportunity to characterize such pathological µcalcifications giving not only their chemical composition but also their spatial distribution in the tissues. This experimental approach offers new opportunities to the clinicians to describe at the cell level the physico-chemical processes leading to the formation of the pathological calcifications which lead to ESRF.

## Introduction

Chronic renal failure is increasing in most industrialized countries as a consequence of acquired systemic diseases such as type II diabetes, which is now the first cause for end stage renal failure [1]. A number of other causes may be responsible for the loss of kidney function and tubular interstitial nephritis, but they are less frequent [2], [3]. Among them, lithogenic diseases may induce intratubular crystallization, which may finally result in end-stage renal failure (ESRF). The diagnosis of such pathological conditions is of a prime importance before kidney transplantation in order to treat efficiently the disease and protect the grafted kidney against recurrence of crystallization. Unfortunately, such rare diseases are often misdiagnosed. The main consequence in affected patients is the progressive degradation of the kidney function which ends up in dialysis [4]. Often, crystals are found in kidney biopsies performed in order to understand the mechanism of the loss of renal function. However, only few histochemical tests are available to attempt an identification of the crystals. Moreover, in some cases, common crystals such as calcium oxalate monohydrate may be present as a consequence of renal failure, but they are not involved in the kidney loss. For these reasons, it is of clinical importance to accurately identify crystals found in the tissue as they can help to early characterization of a disease, which may be efficiently treated by specific drugs. To the best of our knowledge, very few papers have focussed on such subject and only few crystalline phases have been already reported [5], [6].

The aim of this work is to emphasize the chemical diversity of ectopic calcifications present in kidney tissue. In some cases, crystals in tissues are very tiny and classical FTIR microscopy is not sensitive enough to identify their chemical composition. In those cases, Synchrotron Radiation–Fourier Transform Infrared microspectroscopy (µSR-FTIR) can be performed, such technique being able to collect infrared spectra on microscopic-sized minerals present in biopsies. Combined with optical microscopic and raster scanning, chemical cartography obtained with SR- spectroscopy can be associated to an optical image. This experimental configuration allowed us to study different biopsies. Such information regarding the chemical composition of ectopic calcifications will provide insight into the mechanisms leading to the loss of the kidney function.

## Materials and Methods

### Samples

Twenty-four kidney biopsies were investigated. The biological samples came from Necker Hospital (Paris- France). Five microns slices of the biopsies were deposited on low-e microscope slides (MirrIR, Kevley Technologies, Tienta Sciences, Indianapolis). For tissue embedded in paraffin, the paraffin was chemically removed in order to improve the crystal detection under the microscope. Ethical approval was obtained by the ethical committee of Necker Hospital for this study. Each sample was only named by a study number, without indication of the name of the patient or potential identification data. The ethical committee of Necker Hospital had approved this consent procedure.

### Synchrotron FTIR microspectroscopy

The FTIR measurements were carried out at SOLEIL-Synchrotron (St Aubin-Gif sur Yvette, France) on the SMIS beamline [7]. The IR microspectroscopic mappings were collected in reflection mode using an Infrared microscope (Nicplan- Thermo Nicolet) coupled to a FTIR spectrometer (Magma 550-Thermo-Nicolet). The IR microscope is equipped with a motorized sample stage (precision 1 µm) and a liquid nitrogen cooled mercury cadmium telluride (MCT- 250 µm) detector. Most of the analysis and maps presented here were achieved with a projected area on the sample of 6×6 µm^ 2^ and a step size of 6 µm, and each spectrum was acquired after 64 accumulations at 8 cm^−1^ spectral resolution. Data acquisition and processing was performed using Omnic software (Version 7.4, Thermo-Scientific). The compounds were identified by comparing them to reference spectra [8].

## Results

**Figure 1:**
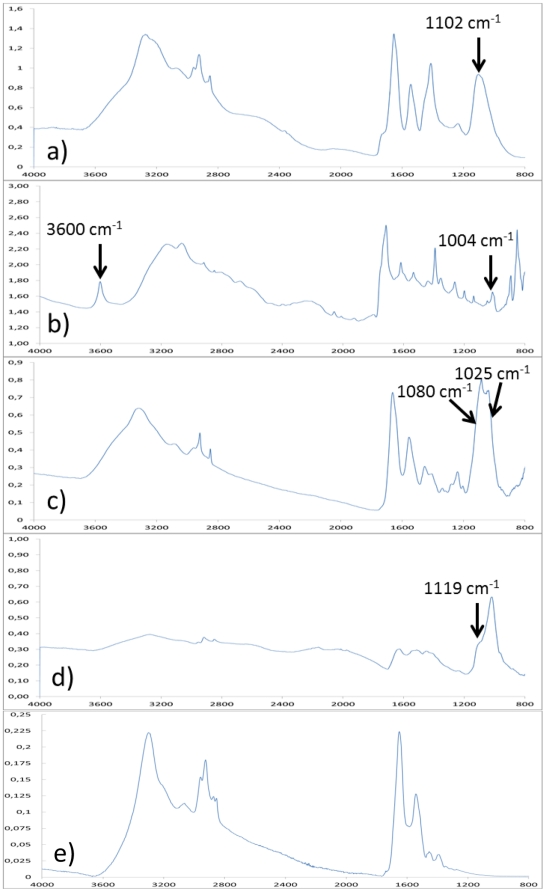
Selected examples of infrared spectra from biopsies. a) Amorphous silica identified by a band at 1102 cm^−1^, b) sodium hydrogen urate monohydrate identified by specific bands at 3600 and 1004 cm^−1^, c) several calcium phosphates including whitlockite (peaks at 1080, 1025 cm^−1^ and associated shoulders, d) octacalcium phosphate and carbapatite, identified by a shoulder at 1119 cm^−1^, e) normal tissue, with signal of water (3300 cm^−1^ and peaks around 1600 cm^−1^) and proteins (peaks at 2900 cm^−1^).

Selected examples of infrared spectra from biopsies are presented in Figure 1. Due to the high brilliance of the synchrotron source, the signal to noise ratio is excellent. Taking advantage of the small size of the probe, it was possible to select a small area of the biopsy for infrared spectra collection and thus to obtain a high quality spectrum of the crystals. Several chemical phases have been identified including amorphous silica through its infrared band at 1102 cm^−1^ (Figure 1a), sodium hydrogen urate monohydrate through its specific bands at 3600 and 1004 cm^−1^ (Figure 1b), and several calcium phosphates including whitlockite (peaks at 1080, 1025 cm^−1^ and associated shoulders, Figure 1c), octacalcium phosphate and carbapatite (Figure 1d).

All the results are gathered in Table 1. Twelve crystalline species were identified and in two cases, precipitates of proteins in tubular lumens were observed. Among crystalline compounds, calcium oxalate was observed commonly as whewellite and in one case as weddellite, which was found as a minor phase in a biopsy containing large amounts of whewellite. Several calcium phosphates were identified: the most common phase was carbapatite. Amorphous carbonated calcium phosphate (ACCP), octacalcium phosphate pentahydrate (OCP) and whitlockite were observed more scarcely. In one sample, some crystals of calcite were observed in association with whewellite. Among the other crystalline species, several purines were identified: sodium hydrogen urate monohydrate, dihydroxyadenine and methyl-1 uric acid. Lastly, foscarnet, a drug used against cytomegalovirus infection, was found in glomeruli in one patient.

**Table 1:** Different chemical phases identified in this study.

Samples	Sex–age (years)	Clinical data	Chemical phase identified
B85	M - 33	Acute renal failure (ARF)	Methyl-1 uric acid
B162	M - 72	Chronic renal failure (CRF)	Whewellite >>Weddellite
B163	M - 72	End stage renal failure (ESRF)	Whewellite
B164	M	Renal papilla	Carbapatite >> amorphous carbonated calcium phosphate (ACCP)
B165	M	Renal papilla	Sodium hydrogen urate monohydrate, Carbapatite and Whitlockite
B166	F - 53	CRF	Dihydroxyadenine
B167	M	ARF	Whewellite
B170	F - 39	CRF	Proteins (amylose)
B171	M - 58	CRF	Whewellite
B173	M - 48	CRF	Carbapatite+whewellite
B174	M - 50	ARF	Proteins
B175	M - 73	CRF, hypercalcemia	Octacalcium phosphate pentahydrate
B176	M - 66	CRF	Whewellite
B177	F - 39	Diabetes - CRF	Silicium dioxide (amorphous Silica)
B178	M - 55	ARF	Dihydroxyadenine
B179	M - 48	ESRF = > Kidney transplantation (KT) = > CRF	Dihydroxyadenine
B180*	M – 51	ESRF = > KT = > CRF	Dihydroxyadenine
B181**	M - 51	ESRF = > KT = > CRF	Dihydroxyadenine
B182	M - 26	ESRF = > KT = > CRF	Carbapatite and octacalcium phosphate pentahydrate
B183	F - 52	Diabetes = > ESRF = > KT = > CMV infection = >ARF	Foscarnet and carbapatite
B184	M - 40	ESRF = > KT	Whewellite
B189	F	Diabetes = > ESRF = > KT = > CRF	Whewellite
B191	M - 74	ESRF = > KT = > CRF	Calcite, whewellite
B192	F - 68	CRF, hypercalcemia	Carbapatite, amorphous calcium phosphate

*Two years after kidney transplantation.

**Five years after kidney transplantation; same patient as for B180. Both biopsies were received in the same time.

**Figure 2:**
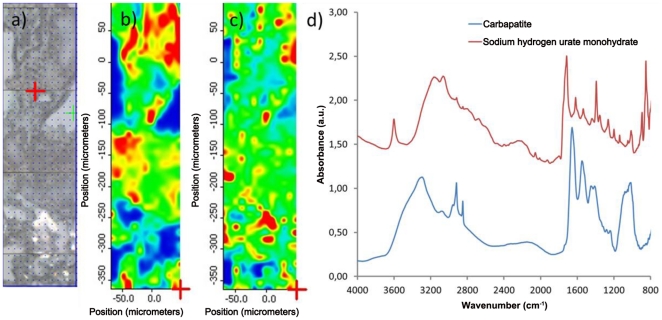
Optical image and mapping of BR165 biopsy (scale from blue to red with increasing concentration), and FT-IR spectra of crystals. a) Optical image of BR165, b) carbapatite map (done at 1030 cm^-1^), c) sodium hydrogen urate monohydrate map (done at 3600 cm^-1^), d) FT-IR spectra of those compounds.

By performing infrared mapping of selected areas of the biopsy, we were able to identify at least 2 different crystalline species in the same sample for 7 biopsies (27%), example in Figure 2 shows a representative example and even three compounds in one case.

**Figure 3:**
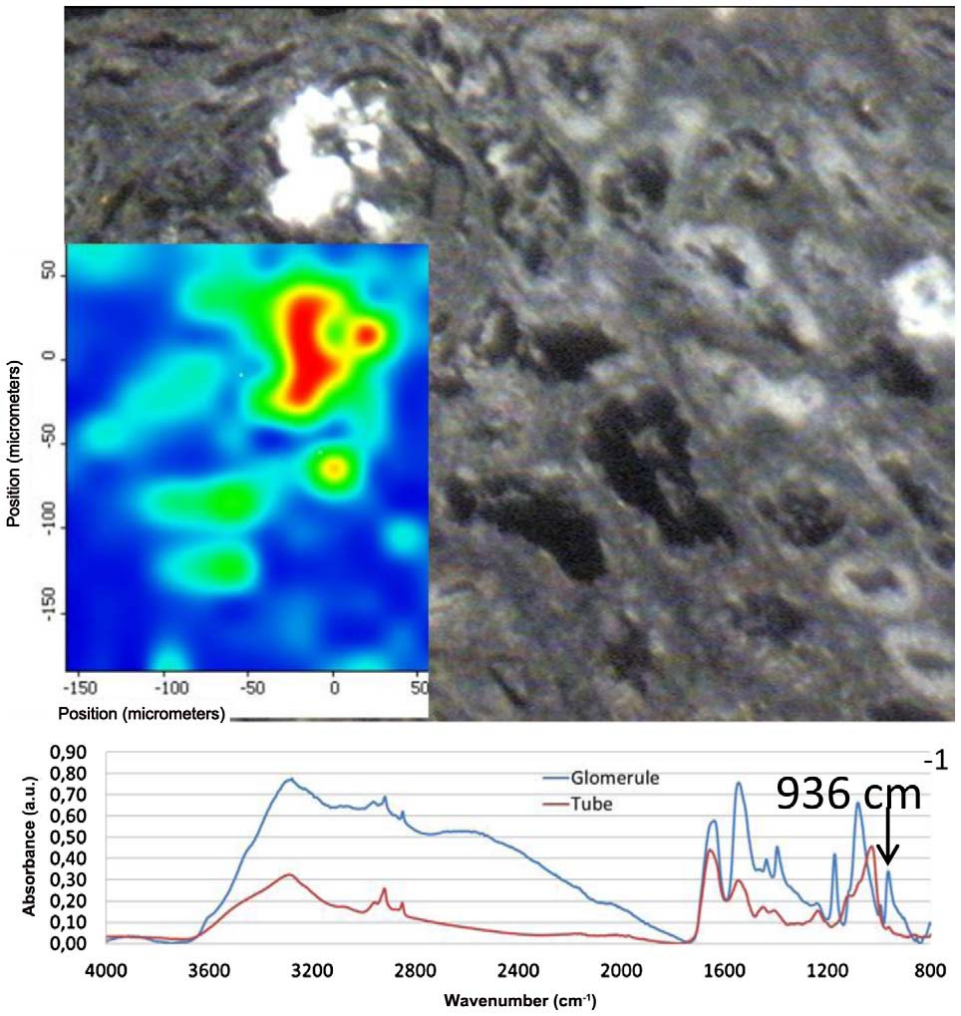
Mapping and optical image of a birefringent structure. The poorly soluble foscarnet can be detected and quantified after a mapping of the biopsy thanks to the characteristic peak at 936 cm^-1^.

Finally, in Figure 3, an optical image of a kidney biopsy shows the glomerulus containing very refringent crystals and the proximal tubules close to the glomerulus, in which pathological deposits were also observed (Fig. 3a). The small size of the probe allowed us to characterize separately the phases deposited in the glomerulus and in the wall of the tubules. The crystals agglomerated in the glomerulus were identified as sodium foscarnet (Fig. 3b). In contrast, deposits within in the cells of the proximal tubules were made of apatite (Fig. 3c).

## Discussion

Crystal deposits within the kidney tissue may occur as a consequence of genetic disorders such as primary hyperoxaluria, adenine phosphoribosyltransferase deficiency, distal renal tubular acidosis, Dent's disease, acquired diseases such as primary hyperparathyroidism, Sjögren's syndrome or intoxication with ethylene glycol which leads to renal failure due to calcium salts deposition.

Classical characterisation of intratissular calcification performed at the hospital is based on tissue coloration with von Kossa and alizarin dyes. These methods help to identified Ca^2+^ oxalate and Ca^2+^ phosphate deposits but are not able to separate whewellite and weddellite or the different Ca^2+^ phosphates namely whitlockite, brushite or apatite.

Through classical FTIR investigation of 25 biopsies, Estepa-Maurice, et al. have underlined the presence of seven chemical phases (whewellite, weddellite, carbapatite, anhydrous uric acid, brushite, dihydroxyadenine and 4′-hydroxytriamterene sulfate) [6]. Thanks to the high flux of synchrotron radiation, micrometer scale calcifications have been characterized. This technique was especially required for analysis of very small crystals (less than 10 µm). Previous experiments [6] were performed on larger crystals (more than 15 µm). Another advantage of synchrotron radiation was its very high signal-to-noise ratio, since such measurements could be the base for a medical diagnosis. Samples could be sent to synchrotrons and that once specific markers are found, then benchtop spectrometers can be used for next measurements. A striking point of our investigation comes from the fact that six new chemical phases have been identified including amorphous silica, sodium hydrogen urate, methyl-1 uric acid and three different Ca^2+^ phosphates namely whitlockite, OCP and ACCP. Moreover, for the first time, mappings with a 10×10 µm^2^ probe have allowed us to underline the chemical heterogeneity of intratissular calcifications. Among the 24 samples which corresponded to 23 patients (B180 and B181 samples correspond to two successive biopsies for the same patient performed two and five years after kidney transplantation), 8 samples contained two to three different crystalline phases.

For the Ca^2+^ phosphates we have already underlined the clinical interest to distinguish the different crystalline species in kidney stones. For example, whitlockite in kidney stones is an infrequent component and has been associated with chronic urinary tract infection in most calculi in women [9]. In contrast, brushite was often associated with hypercalciuria [10] and primary hyperparathyroidism [11]. The presence of OCP is quite interesting. OCP is a thermodynamically metastable phase and it transforms to apatite spontaneously [12]. From a physiological point of view, this compound has been proposed to participate as a precursor of biological apatites. Also, regarding kidney stones, this compound has been found in pregnant women for which the metabolism of Ca^2+^ is modified in order to build the bone network of the foetus [13]. For these reasons, it seems of primary importance to distinguish these different Ca^2+^ phosphates in the tissues.

Another original aspect of our study comes from the opportunity to use SR µFTIR spectroscopy for indirect diagnosis of a genetic disease. Dihydroxyadenine crystals were found in four patients (five biopsies in Table 1). Such crystals deposits in parenchyma are pathognomonic of a rare disease, adenine phosphoribosyltransferase deficiency, an inherited disease able to induce recurrent kidney stones and/or kidney failure. Dihydroxyadenine is often too late identified in patients who have developed renal insufficiency and sometimes after the crystal-induced destruction of a kidney transplant. As recently emphasized, these data suggest this disease could be less rare than commonly reported [14]. In our series, two patients were diagnosed using synchrotron examination of biopsies after renal impairment of the grafted kidney. They were treated with allopurinol and they recovered a large part of their kidney function. Such observations underline the clinical interest of early identification of crystals in the tissue.

Finally, high spatial resolution infrared mapping is a powerful tool to study the kidney distribution of some poorly soluble drugs and metabolites. It is well known that foscarnet crystallizes in glomeruli [15]. Foscarnet (phosphonoformic acid) is a pyrophosphate analogue that inhibits the DNA polymerase of all herpes viruses, which is commonly used in immuno-suppressed patients (AIDS, grafted patients) who developed CMV infection. Through SR-µ FTIR, spectroscopy we observed sodium foscarnet crystals in glomeruli in line with a previous study [16]. In addition, we found in the cells of the proximal tubule an accumulation of apatite crystals suggesting that the drug is locally metabolized by splitting the bond between the two phosphate residues of foscarnet. To the best of our knowledge, no data has been reported about associated crystalline phases deposited within the cells of the tubules.

In conclusion, here, we give the first structural evidence of a chemical diversity for mineral deposits in the kidney tissue. New crystalline phases were described such as amorphous silica, sodium hydrogen urate, methyl-1 uric acid and three different Ca^2+^ phosphates namely whitlockite, OCP and ACCP. Moreover, for the first time, we underline the chemical heterogeneity of intratissular calcifications.

Other striking results of this study concern the opportunity to establish early medical diagnosis. Such approach should be generalized when the usual techniques at the hospital are not able to identify accurately pathological intratissular deposits. The relatively high number of APRT deficiency cases identified from dihydroxyadenine crystals, a disease easily treated with allopurinol when correctly diagnosed, underlined the utmost clinical importance of identifying crystals in kidney biopsies in order to improve the etiologic diagnosis of some renal impairments.
